# Internet use for pregnancy-related information and its correlates among women attending antenatal care in Mogadishu, Somalia

**DOI:** 10.1371/journal.pdig.0001590

**Published:** 2026-07-30

**Authors:** Mohamed Mustaf Ahmed, Luul Mohamed Ibrahim, Roodo Abdullahi Elmi, Zhinya Kawa Othman, Francesco Branda, Olalekan John Okesanya, Uthman Okikiola Adebayo, Shuaibu Saidu Musa, Abdullahi Hassan Elmi, Abdirasak Sharif Ali, Don Eliseo Lucero-Prisno III

**Affiliations:** 1 Faculty of Medicine and Health Sciences, SIMAD University, Mogadishu, Somalia; 2 Department of Pharmacy, Kurdistan Technical Institute, Sulaymaniyah, Kurdistan Region, Iraq; 3 Unit of Medical Statistics and Molecular Epidemiology, Campus Bio-Medico University of Rome, Rome, Italy; 4 Department of Public Health and Maritime Transport, University of Thessaly, Volos, Greece; 5 Department of Medical Laboratory Science, Neuropsychiatric Hospital, Aro, Abeokuta, Nigeria; 6 Department of Medical Laboratory Science, Federal University of Health Sciences Teaching Hospital, Ila-Orangun, Nigeria; 7 Department of Medical Laboratory Science, College of Basic Health Sciences, Achievers University, Owo, Nigeria; 8 School of Global Health, Faculty of Medicine, Chulalongkorn University, Bangkok, Thailand; 9 Department of Nursing, Dr. Sumait Hospital, Faculty of Medicine and Health Sciences, SIMAD University, Mogadishu, Somalia; 10 Department of Microbiology and Laboratory Sciences, Faculty of Medicine and Health Sciences, SIMAD University, Mogadishu, Somalia; 11 Department of Global Health and Development, London School of Hygiene and Tropical Medicine, London, United Kingdom; 12 Center for Research and Development, Cebu Normal University, Cebu, Philippines; 13 Research Unit, Bukidnon State University, Malaybalay City, Bukidnon, Philippines; University of Pittsburgh School of Medicine, UNITED STATES OF AMERICA

## Abstract

Somalia faces persistent gaps in maternal health services, while digital connectivity is expanding rapidly, positioning online platforms as potential complements to antenatal counselling. This study assessed how often pregnant women attending antenatal care in Somalia’s capital, Mogadishu, use the internet for pregnancy-related information and examined the sociodemographic and clinical factors associated with that behaviour. We conducted an analytical cross-sectional study at six antenatal clinics in Mogadishu from February to May 2025, in which eligible literate pregnant women completed a structured, self-administered questionnaire, and we used multivariable logistic regression to identify factors associated with internet use. Among the 422 participants, 78.2% reported using the internet to obtain pregnancy-related information during the current pregnancy. Daily use predominated, social media was the primary means of access, and seeking support was the most frequently reported reason. Commonly searched topics included diet during pregnancy, fetal development, and infant feeding. Higher monthly income, being in the third trimester, and reporting a current pregnancy-related health problem were each associated with greater odds of internet use, whereas age, education, employment, number of previous pregnancies, number of living children, and reported fetal sex were not. Among these literate women attending antenatal care in Mogadishu, the internet, mainly through social media, is already a common source of pregnancy-related information, embedded in their everyday information practices. Integrating trustworthy Somali-language content and clinician-linked channels into antenatal care therefore warrants prospective evaluation, alongside attention to digital literacy, exposure to misinformation, privacy, and maternal and newborn outcomes. Because the study was cross-sectional and clinic-based, the findings describe associations rather than causation and should not be generalized to all pregnant women in Mogadishu.

## Author summary

Pregnant women increasingly turn to the internet to answer everyday questions about pregnancy; however, we know little about how women in Somalia do this. Somalia has some of the world’s highest maternal death rates, and at the same time, internet and social media use is growing quickly. Therefore, it is important to determine whether women are using online tools to fill the gaps in the care they receive. We surveyed 422 pregnant women who attended six antenatal clinics in Mogadishu between February and May 2025. We found that roughly four in five women had used the internet for pregnancy information during their current pregnancy, most of them daily, and social media was the most common source. Women most often looked for information on diet, baby development, and infant feeding. Women with higher incomes, those further along in their pregnancy, and those with current health concerns were more likely to search online. Our findings suggest that trustworthy Somali-language information should be incorporated into routine antenatal care.

## 1. Introduction

Somalia faces persistent maternal health challenges alongside fast-changing digital access, a combination that makes understanding online information-seeking during pregnancy both timely and relevant to policy [1,2]. Recent UN and World Bank estimates place Somalia’s maternal mortality ratio at 563 deaths per 100,000 live births in 2023, which is one of the highest globally [3]. National survey–based analyses report low service coverage across the continuum of care, including roughly 31% of women receiving any antenatal care, about 24% completing four or more visits, and about 21% delivering in a health facility [4]. Although these indicators originate from different data systems and reference periods, they consistently describe substantial maternal service gaps in Somalia rather than a single harmonized trend estimate. These gaps occur in a youthful population, in which nearly half are children aged 0–14 years, and in a health system repeatedly strained by displacement, insecurity, and resource limitations, as documented by the WHO and its partners. Simultaneously, connectivity is expanding [5].

Official statistics indicate that 28% of Somalis used the internet in 2022, based on World Bank estimates [6], where internet use refers to individuals who accessed the internet from any location during the previous three months. Complementing this, industry reporting at the start of 2025 described approximately 10.7 million internet users, 55.2% internet penetration, and about 3.05 million social media user identities in Somalia, signaling rapid digital expansion in everyday life [7]. Because these indicators rely on different definitions and denominators, they are interpreted here as complementary evidence of digital growth [8]. Infrastructure developments continue apace, with the government licensing Starlink in April 2025 to extend broadband to underserved areas [9]. In low- and middle-income settings, pregnant women commonly supplement clinical counselling with information from websites, apps, and social media platforms [10–12]. Studies have shown a high uptake of online pregnancy information, frequent use early in gestation, and growing reliance on social media communities for peer support [13–15]. Studies suggest that digital and messaging interventions have been linked to improved knowledge and antenatal engagement in some contexts, including WhatsApp-based and tailored mobile health approaches in sub-Saharan Africa [16,17].

**Fig 1:**
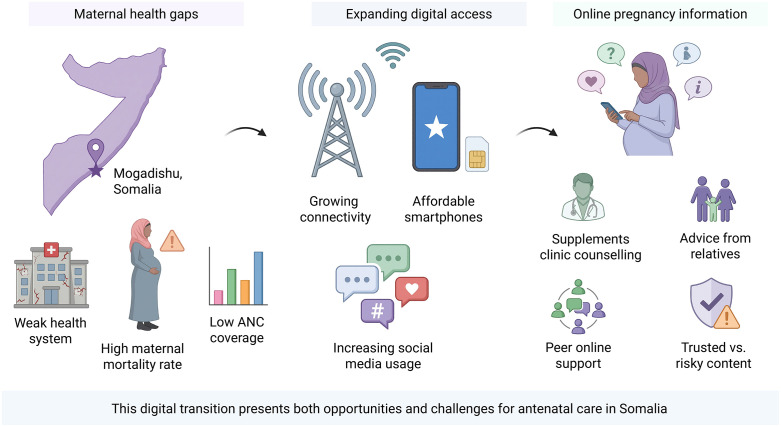
Maternal health in Mogadishu, Somalia: gaps, digital access, and online pregnancy information. Created in BioRender. Ahmed, M. M. (2026) https://BioRender.com/rpy40y7.

However, these benefits depend on information quality and user e-health literacy. Evaluations repeatedly document variability in the readability and accuracy of pregnancy content and uneven quality in maternal health apps, while broader evidence highlights the risks of misinformation and anxiety linked to unmoderated social feeds [18,19]. Within Somalia, policy and research communities increasingly emphasize the potential of digital health; however, published evidence describing how antenatal clinic attendees use the internet for pregnancy-related information and which sociodemographic or obstetric factors are associated with that behavior remains limited relative to the scale of maternal health needs [2,20]. Accordingly, this study focuses on pregnant women attending antenatal care in selected Mogadishu clinics, and the findings are intended to represent this clinic-attending group rather than all pregnant women in Mogadishu or Somalia. Within this clinic-attending population, we estimated the prevalence of internet use for pregnancy-related information during the current pregnancy, described channels and patterns of use, and identified associated factors. This study advances current low- and middle-income country (LMIC) evidence by providing setting-specific data from urban Somalia that can inform ANC-linked counselling and locally appropriate digital health planning while reserving causal and population-wide inferences for future longitudinal and population-based research (Fig 1).

## 2. Methods

### 2.1 Study design and setting

We conducted an analytical cross-sectional study in Mogadishu, Somalia, from February 2025 to May 2025. The setting comprised six antenatal clinics (public and private) across the Banaadir region, where antenatal care (ANC) is the most common first point of contact with the health-care system.

### 2.2 Population, eligibility, and sampling

**Fig 2:**
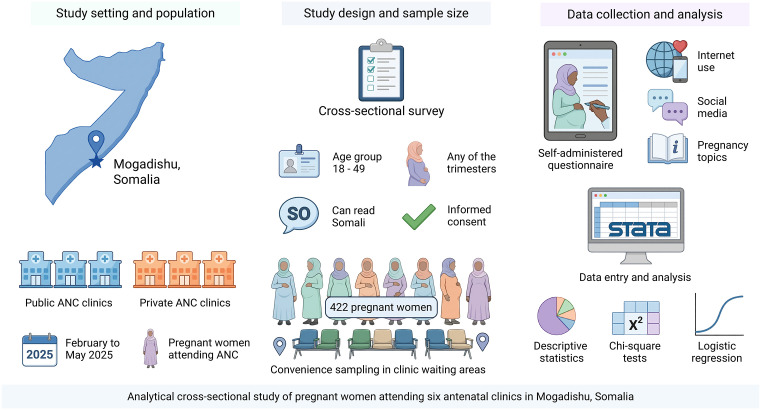
Study setting, design, and sampling of internet use for pregnancy information in Mogadishu. Created in BioRender. Ahmed, M. M. (2026) https://BioRender.com/jiiek6k.

The target population was pregnant women in any trimester who attended the selected clinics during the study period (Fig 2). The inclusion criteria were age 18–49 years, attendance at ANC on the survey days, ability to read and provide informed consent, and fluency in the Somali language. We excluded women who were unable to read or write, or those who were too unwell to participate. A non-probability clinic-based sampling approach was used, with the consecutive recruitment of eligible attendees until the daily quota per facility was met. Because participation required literacy and clinic attendance, the sample likely under-represented women with lower literacy and those not engaged in ANC, who may differ in socioeconomic position and internet access. Therefore, prevalence and association estimates are interpreted for this clinic-attending, literate population and not as population-representative estimates for all pregnant women in Mogadishu, Somalia.

This sampling frame aligns with the study objective of characterizing internet-based pregnancy information-seeking among women able to engage directly with online content and complete a self-administered questionnaire. Because women with lower literacy often face structural barriers to smartphone and internet access, their digital information behaviors may differ substantially; accordingly, prevalence and correlates are estimated within this clinic-attending, literate ANC population rather than for all pregnant women in Mogadishu or Somalia.

### 2.3 Sample size determination

The sample size for this study was calculated using Cochran’s formula to estimate the population proportion with a specified margin of error. This method is appropriate for studies with large or unknown population sizes and is widely used in epidemiological studies. The formula is expressed as follows:


$$\begin{array}{c} n=\frac{z^{\text{2}}\;p\;(\text{1}-p)}{e^{\text{2}}} \end{array}$$

where n represents the required sample size, z denotes the z-score corresponding to the desired confidence level, p is the estimated proportion of the attribute in the population, and e signifies the desired margin of error.

In this study, the following parameters were applied: z = 1.96 (corresponding to a 95% confidence level), p = 0.5 (assuming maximum variability), and e = 0.05 (5% margin of error). To account for the maximum variability due to the unknown proportion of internet use among pregnant women, a proportion of p = 0.5 was assumed in Cochran’s formula, resulting in an initial sample size of approximately 384 participants. To account for potential non-response, the sample size was increased by 10%, yielding a final required sample size of 422.

### 2.4 Instrument, translation, and pilot testing

Data were collected using a structured, self-administered questionnaire adapted from a validated tool used among pregnant women attending primary health-care centers in Qatar [20], with contextual modifications for use in Somalia. Forward translation into Somali and independent back-translation were performed to ensure semantic equivalence. The content validity was reviewed by three local maternal health clinicians and three public health researchers. A pilot study with 30 pregnant women assessed clarity, cultural appropriateness, and administration time, followed by minor wording changes. Specifically, the revisions included simplifying item phrasing, replacing ambiguous terms with locally familiar Somali wording, standardizing response options, and clarifying instructions for single-response versus multiple-response items. The test–retest reliability of the subsample supported the stability of the key items.

### 2.5 Data collection and statistical analysis

The questionnaires were completed in private areas of the clinic’s waiting rooms after written informed consent was obtained. Trained female data collectors explained the study aims, answered questions, and checked the forms for completeness without influencing responses. Analyses were conducted using Stata (StataCorp), version 19.5. The primary outcome was internet use during the current pregnancy (yes vs. no). To maintain consistency with the questionnaire structure, the “main purpose of searching” among users (support, sharing experiences, or looking for pregnancy-related information) was analyzed as a separate pattern-of-use variable and was not used to redefine the primary binary outcome. The prevalence of internet use was calculated as p = x/n, where x is the number of participants reporting any such use and n is the total number of respondents.

Explanatory variables were prespecified a priori based on a conceptual framework and prior literature [21], and coded as categorical factors: age (18–25, 26–35, ≥36 years), highest education (no formal education, primary, secondary, university), employment (employed, housewife, student), monthly income (≤100 USD, 101–300 USD, >300 USD), gravida (primigravida, multigravida), number of living children (no children, 1–3, ≥4), trimester (first, second, third), reported fetal gender at the time of survey (female, male, prefer not to say/don’t know). The ‘prefer not to say/don’t know’ category was coded as an observed response category (reflecting uncertainty or non-disclosure at the time of the survey), not as item non-response. Any health problem during the current pregnancy was coded as yes or no. Health problems during the current pregnancy were defined as presence of at least one listed self-reported condition or symptom during the current pregnancy (e.g., low back pain, vomiting, anemia, gestational diabetes, morning sickness, heartburn, or other). Multiple responses were permitted; therefore, category-specific percentages were not mutually exclusive, and these items were not clinically adjudicated diagnoses. For regression, the reference categories were 18–25 years, no formal education, employed, ≤100 USD, primigravida, no children, first trimester, female fetal gender, and no health problems. Data completeness was checked for all variables used in the analysis. In the final dataset, there were no missing values for the variables included in the descriptive and multivariable analyses; therefore, all participants were retained in the regression model.

Age and monthly income were analyzed using prespecified categories to support interpretability and comparability with prior maternal health reporting (age: 18–25, 26–35, and ≥36 years; monthly income: ≤100 USD, 101–300 USD, and >300 USD). Category boundaries were defined a priori based on literature review relevance and the distribution of responses. Descriptive summaries of continuous values are provided (mean ± standard deviation [SD] or median [interquartile range, IQR]) for context. Bivariate associations between internet use and covariates were examined using Pearson’s chi-square test. Covariates were primarily prespecified a priori based on the conceptual framework and prior literature. In line with prior practice and methodological recommendations, we used a liberal bivariable screening threshold (p < 0.25) as a supplementary step to avoid excluding potentially important covariates before multivariable modeling (common screening range: p < 0.20–0.25) [22,23]. Adjusted odds ratios (AORs) with 95% confidence intervals were estimated for each covariate. The final model was estimated using standard model-based standard errors, and the results are presented as AORs with 95% confidence intervals and p-values (reported to three decimal places). Accordingly, inferential statistics are presented for full reporting, but findings are interpreted as association estimates within this clinic-attending sample.

As participants were recruited from six clinics, their responses may have been correlated within the facilities. Therefore, standard errors may be underestimated if within-clinic correlation is present. With only six clusters, standard cluster-robust standard errors, GEE, and mixed-effects models with random clinic effects are not reliable corrective options: simulation studies consistently recommend a minimum of approximately 20–50 clusters for cluster-robust inference to be trustworthy, and fitting random-effects models with fewer than ten clusters frequently yields unstable variance estimates [24,25]. Applying these methods with six clusters could therefore introduce spurious precision rather than genuinely correct for dependence. Standard model-based standard errors were therefore retained. All confidence intervals and p-values should be interpreted as approximate, with primary emphasis on the direction and magnitude of associations. Model diagnostics (including multicollinearity and goodness-of-fit checks) were treated as supportive assessments and not as the sole criteria for model adequacy. Model discrimination was assessed using the C-statistic (area under the ROC curve) and calibration using the Hosmer–Lemeshow goodness-of-fit test (10 groups). All tests were two-sided with α = 0.05. Given the high outcome prevalence, the adjusted odds ratios were interpreted as measures of association and may overstate the effect magnitude relative to the prevalence ratios. To address this, a sensitivity analysis was conducted using modified Poisson regression with robust standard errors to estimate adjusted prevalence ratios (APRs) for all covariates. To assess robustness to collinearity between gravida and number of living children, three alternative logistic regression models were fitted: (1) excluding gravida, (2) excluding number of living children, and (3) excluding both.

### 2.6 Ethics Statement

Ethical approval was obtained from the Institutional Review Board of SIMAD University, Mogadishu, Somalia (Ref. 2025/SU-IRB/FMHS/P007; approval date 30 January 2025). Written informed consent was obtained from all participants prior to enrolment. Participants were informed about the study objectives and procedures, the voluntary nature of participation, their right to confidentiality, and their right to withdraw at any time without consequences. All procedures adhered to the recognized ethical principles, including the Declaration of Helsinki, and confidentiality safeguards were maintained throughout the data collection and handling.

## 3. Results

### 3.1 Participant characteristics

A total of 422 pregnant women participated in this study. The mean age was 25.87 years (SD 5.61), and the median age was 25 years (IQR: 22–29). More than half of the participants were aged 18–25 years (56.9%), followed by 26–35 years (37.2%), and 36 years or older (5.9%) (Table 1). Regarding education, 11.1% had no formal schooling, 35.3% had primary education, 35.8% had secondary education, and 17.8% had a university education. Over half were housewives (54.0%), 24.4% were employed, and 21.6% were students. The mean reported monthly income was 274.78 USD (SD 123.33), and the median was 250 USD (IQR 200–400); 9.7% reported less than 100 USD, 62.6% reported 101–300 USD, and 27.7% reported more than 300 USD.

**Table 1:** Sociodemographic characteristics of the participants (N = 422).

Variable	Frequency (%)
**Age (years)**	
18-25	240 (56.87%)
26-35	157 (37.20%)
36 or more	25 (5.92%)
Mean ± SD	25.86 ± 5.60
Median (IQR)	25 (22–29)
**Highest education level**	
No formal education	47 (11.14%)
Primary	149 (35.31%)
Secondary	151 (35.78%)
University	75 (17.77%)
**Employment status**	
Employed	103 (24.41%)
Housewife	228 (54.03%)
Student	91 (21.56%)
**Monthly income (USD)**	
Less than 100	41 (9.72%)
101-300	264 (62.56%)
More than 300	117 (27.7%)
Mean ± SD	274.77 ± 123.33
Median (IQR)	250 (200–400)
**Gravida**	
Primigravida	115 (27.25%)
Multigravida	307 (72.75%)
**Number of living children**	
No children	116 (27.49%)
1–3 children	205 (48.58%)
4 or more children	101 (23.93%)
**Trimester**	
First	65 (15.40%)
Second	187 (44.31%)
Third	170 (40.28%)
**Reported fetal gender**	
Female	166 (39.34%)
Male	113 (26.78%)
Prefer not to say/I don’t know	143 (33.89%)
**Health problems during the current pregnancy**	
No	80 (18.96%)
Yes	342 (81.04%)
Anemia	130 (30.81%)
Low back pain	157 (37.2%)
Gestational diabetes	128 (30.33%)
Vomiting	131 (31.04%)
Morning sickness	109 (25.83%)
Heartburn	86 (20.38%)
Others*	32 (7.5%)

*Other health problems included dizziness, edema, and headaches. Categories were non-mutually exclusive because participants could report more than one health problem.

Most of the participants were multigravida (72.8%). Regarding living children, 27.5% had none, 48.6% had one to three, and 23.9% had four or more. Participants were distributed across trimesters as follows: first, 15.4%; second, 44.3%; and third, 40.3%. Regarding the reported sex of the fetus, 39.3% indicated female, 26.8% male, and 33.9% preferred not to say or did not know. This category reflects participants who either did not know the fetal sex or chose not to disclose it. Health problems during the current pregnancy were reported by 81.0% of participants; in the total sample, low back pain was reported by 37.2%, vomiting by 31.0%, anemia by 30.8%, gestational diabetes by 30.3%, morning sickness by 25.8%, and heartburn by 20.4%, with 7.5% reporting other problems such as dizziness, edema, and headache. These condition-specific estimates reflect self-reported responses to multiple-response items; therefore, the percentages are non-mutually exclusive and may overlap across conditions.

### 3.2 Sources of pregnancy-related information

Most participants identified healthcare providers as a source of information (90.0%), followed by family members (83.2%) and the internet (78.2%); smaller proportions reported friends (30.8%), television (24.4%), and books or magazines (16.1%). Most participants reported participation in educational activities related to pregnancy (91.5%). Among the participants, primary healthcare centers were the most frequently reported venue (43.26%), followed by governmental hospitals (32.64%), private antenatal clinics (17.88%), and private hospitals (6.22%) (Table 2).

**Table 2:** Pregnancy-related information sources (N = 422).

Source	Frequency (%)
**Internet**	
No	92 (21.80%)
Yes	330 (78.20%)
**Healthcare providers**	
No	42 (9.95%)
Yes	380 (90%)
**Family members**	
No	71 (16.82%)
Yes	351 (83.18%)
**Friends**	
No	292 (69.19%)
Yes	130 (30.81%)
**Books or magazines**	
No	354 (83.89%)
Yes	68 (16.11%)
**Television**	
No	319 (75.59%)
Yes	103 (24.41%)
**Participation in educational activity**	
No	36 (8.53%)
Yes	386 (91.47%)
**Place of the educational activity (N = 386)**	
Governmental hospital	126 (32.64%)
Primary healthcare center	167 (43.26%)
Private antenatal clinic	69 (17.88%)
Private hospital	24 (6.22%)

### 3.3 Patterns of internet use among internet users

**Fig 3:**
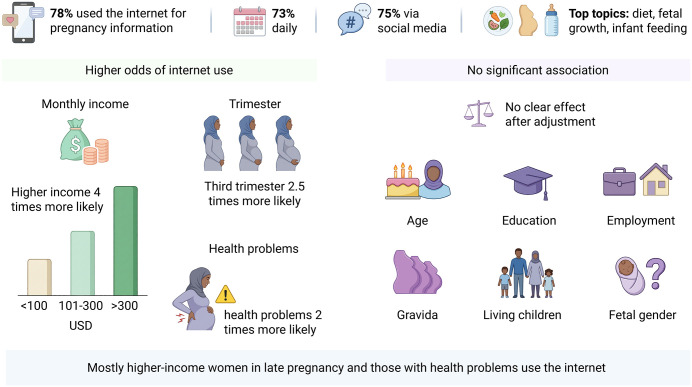
Key findings on internet use for pregnancy information among antenatal clinic attendees. Created in BioRender. Ahmed, M. M. (2026) https://BioRender.com/ior73z8.

Among the 330 participants who reported using the internet for pregnancy-related information, 72.7% did so every day during the current pregnancy, 17.9% a few times during pregnancy, 5.5% one to two times per month, and 3.9% three to four times per week. Social media was the most common method for accessing information (74.9%), followed by mobile applications (23.9%) and websites (1.2%). The main stated purpose of searching was to seek support (70.9%), with additional reports of sharing experiences with others (27.0%) and seeking pregnancy-related information (2.1%) (Table 3). These purpose categories were questionnaire response options among Internet users and are presented descriptively. Overall, the findings indicate that participants mainly accessed pregnancy-related resources through social media and app-based channels, while websites contributed minimally to this sample. The most frequently searched topics were diet during pregnancy (41.5%), fetal development (28.5%), and infant feeding (22.1%), with smaller proportions searching for pregnancy complications, preparation for delivery, infant care, medication use, physical activity during pregnancy, and personal care (Fig 3).

**Table 3:** Patterns of internet use among participants (N = 330).

Variable	Frequency (%)
**Frequency of internet use during the current pregnancy**	
Everyday	240 (72.73%)
3–4 times a week	13 (3.94%)
1–2 times a month	18 (5.45%)
Few times during pregnancy	59 (17.88%)
**Methods used to access pregnancy-related information**	
Social media	247 (74.85%)
Mobile applications	79 (23.94%)
Websites	4 (1.21%)
**Main purpose of searching the internet during pregnancy**	
Looking for support	234 (70.91%)
Sharing experiences with others	89 (26.97%)
Looking for pregnancy-related information	7 (2.12%)

### 3.4 Relationship between participant characteristics and internet use

Overall, 330 of the 422 participants reported using the internet for pregnancy-related information, corresponding to a prevalence of 78.2% (Table 4). In bivariable analyses, the distribution of internet use differed by monthly income, trimester, gender of the fetus, and the presence of health problems during the current pregnancy. Internet use was reported by 61.0% of those with an income less than 100 USD, 77.3% of those with 101–300 USD, and 86.3% of those with more than 300 USD, with a p-value of 0.003. Internet use was reported by 66.2% of participants in the first trimester, 77.5% in the second, and 83.5% in the third, with a p-value of 0.015. By reported fetal gender, the proportion of those using the internet was 84.9% among those reporting female, 75.2% among those reporting male, and 72.7% among those who preferred not to say or did not know (*p* = 0.023). Internet use was reported by 66.3% of participants without health problems and 81.0% of those with health problems (p = 0.004). No statistically significant differences were observed across age group, education level, employment status, gravida, or number of living children in the bivariable analyses.

**Table 4:** Relationship between participant characteristics and use of the internet for pregnancy-related information (N = 422).

Internet use for pregnancy-related information				
**Variable**	**No**	**Yes**	**Total**	**p*-*value**
	92 (21.8%)	330 (78.2%)	422 (100.0%)	
**Age (years)**				
18-25	54 (58.7%)	186 (56.4%)	240 (56.9%)	0.917
26-35	33 (35.9%)	124 (37.6%)	157 (37.2%)	
36 or more	5 (5.4%)	20 (6.1%)	25 (5.9%)	
**Highest education level**				
No formal education	13 (14.1%)	34 (10.3%)	47 (11.1%)	0.581
Primary	35 (38.0%)	114 (34.5%)	149 (35.3%)	
Secondary	30 (32.6%)	121 (36.7%)	151 (35.8%)	
University	14 (15.2%)	61 (18.5%)	75 (17.8%)	
**Employment status**				
Employed	20 (21.7%)	83 (25.2%)	103 (24.4%)	0.596
Housewife	54 (58.7%)	174 (52.7%)	228 (54.0%)	
Student	18 (19.6%)	73 (22.1%)	91 (21.6%)	
**Monthly income (USD)**				
Less than 100	16 (17.4%)	25 (7.6%)	41 (9.7%)	0.003*
101-300	60 (65.2%)	204 (61.8%)	264 (62.6%)	
More than 300	16 (17.4%)	101 (30.6%)	117 (27.7%)	
**Gravida**				
Primigravida	27 (29.3%)	88 (26.7%)	115 (27.3%)	0.610
Multigravida	65 (70.7%)	242 (73.3%)	307 (72.7%)	
**Number of living children**				
No children	26 (28.3%)	90 (27.3%)	116 (27.5%)	0.981
1-3 children	44 (47.8%)	161 (48.8%)	205 (48.6%)	
4 or more children	22 (23.9%)	79 (23.9%)	101 (23.9%)	
**Trimester**				
First	22 (23.9%)	43 (13.0%)	65 (15.4%)	0.015*
Second	42 (45.7%)	145 (43.9%)	187 (44.3%)	
Third	28 (30.4%)	142 (43.0%)	170 (40.3%)	
**Reported fetal gender**				
Female	25 (27.2%)	141 (42.7%)	166 (39.3%)	0.023*
Male	28 (30.4%)	85 (25.8%)	113 (26.8%)	
Prefer not to say/don’t know	39 (42.4%)	104 (31.5%)	143 (33.9%)	
**Health problems during the current pregnancy**				
No	27 (29.3%)	53 (16.1%)	80 (19.0%)	0.004*
Yes	65 (70.7%)	277 (83.9%)	342 (81.0%)	

Note. p-values are from Pearson’s chi-square test; an asterisk (*) indicates statistical significance at p < 0.05 (two-sided).

### 3.5 Multivariable analysis

In the multivariable logistic regression, higher monthly income remained associated with reporting internet use for pregnancy-related information after adjustment for covariates. Compared with less than 100 USD, the adjusted odds ratio was 2.15 (95% confidence interval: 1.02–4.51) for 101–300 USD and 3.83 (95% confidence interval: 1.60–9.17) for > 300 USD (Table 5). Third-trimester women had higher odds of using the internet than first-trimester women, with an adjusted odds ratio of 2.49 and a 95% confidence interval of 1.24–5.03. Reporting a health problem during the current pregnancy was also associated with higher odds of internet use compared with no health problem (adjusted odds ratio 2.03, 95% confidence interval 1.15–3.60). No statistically significant associations were observed between age group, education level, employment status, gravida, number of living children, or reported gender of the fetus in the adjusted model (Table 5). The C-statistic (area under the ROC curve) for the multivariable model was 0.689, indicating moderate discrimination. The Hosmer–Lemeshow goodness-of-fit test (10 groups) yielded χ²  =  1.90, df  =  8, p  =  0.984, indicating adequate model calibration. These diagnostics are reported as supportive summaries and are interpreted cautiously given the small number of clusters and the cross-sectional design. A sensitivity analysis using modified Poisson regression with robust standard errors produced APRs that were directionally consistent with the AOR estimates but attenuated in magnitude, as expected when outcome prevalence is high (S1 Table). Three additional logistic regression models examining robustness to collinearity between gravida and number of living children, excluding each variable individually and both simultaneously, yielded stable estimates for the three primary associations (monthly income, third trimester, current health problems) across all specifications (S2 Table).

**Table 5:** Factors associated with use of the internet for pregnancy-related information (N = 422).

Variable	Adjusted OR (95% CI)	p-value
**Age (years)**		
18–25	1.00	
26–35	1.14 (0.62, 2.11)	0.668
36 or more	1.49 (0.43, 5.11)	0.530
**Highest education level**		
No formal education	1.00	
Primary	1.16 (0.52, 2.59)	0.716
Secondary	1.58 (0.69, 3.62)	0.281
University	1.45 (0.56, 3.79)	0.445
**Employment status**		
Employed	1.00	
Housewife	0.93 (0.49, 1.77)	0.830
Student	1.32 (0.59, 2.92)	0.500
**Monthly income (USD)**		
< 100	1.00	
101–300	2.15 (1.02, 4.51)	0.043*
More than 300	3.83 (1.60, 9.17)	0.003*
**Gravida**		
Primigravida	1.00	
Multigravida	1.50 (0.33, 6.77)	0.599
**Number of living children**		
No children	1.00	
1–3 children	0.57 (0.13, 2.59)	0.468
4 or more children	0.43 (0.08, 2.21)	0.314
**Trimester**		
First	1.00	
Second	1.76 (0.90, 3.43)	0.098
Third	2.49 (1.24, 5.03)	0.011*
**Reported fetal gender**		
Female	1.00	
Male	0.61 (0.32, 1.18)	0.144
Prefer not to say / Don’t know	0.65 (0.36, 1.20)	0.170
**Health problems during current pregnancy**		
No	1.00	
Yes	2.03 (1.15, 3.60)	0.017*

Note. Adjusted odds ratios (AOR) with 95% confidence intervals and p-values are from multivariable logistic regression; an asterisk (*) indicates statistical significance at p < 0.05 (two-sided).

## 4. Discussion

This study among antenatal clinic attendees in Mogadishu found that internet use during pregnancy is common and occurred predominantly through social media rather than websites. In this setting, the dominant pattern reflects social media–mediated support and experience sharing, whereas website-based use is uncommon. Our primary outcome captured any internet use during the current pregnancy (yes/no); intensity of engagement and main purpose (support-seeking, experience-sharing, information-seeking) were examined separately as patterns of use among users. Patterned differences emerged across socioeconomic and clinical characteristics, with greater use among those in later gestation, respondents reporting current pregnancy health problems, and across household income categories. Taken together, these findings indicate that women in this clinic-attending urban sample are integrating digital sources into pregnancy-related information ecosystems. International evidence similarly shows that many pregnant women supplement clinical counselling with internet platforms, apps, and social media to meet their informational and psychosocial needs across gestation [10,13,26].

The fact that the modal access pathway in our sample was social media is also consistent with studies showing that peer communities are perceived as timely, relatable, and available after hours, and that they often serve the dual role of providing information and social support [27–29]. The gestational-stage pattern we observed, with greater reported use later in pregnancy, has been noted in some contexts and contrasts with others, underscoring that the timing of online engagement can be shaped by local care pathways, content availability, and perceived risks in each trimester. In Qatar, whose instrument informed our questionnaire adaptation, authors reported higher online use among women later in pregnancy, whereas studies from East Asia and parts of Europe identified surges earlier in gestation when uncertainty is high and first appointments may be delayed [13,21,30–32]. These competing patterns suggest that the cadence of antenatal contacts and the structure of health systems influence when people turn to the internet for information on pregnancy. In interpreting the association with health problems, these reports reflect self-reported rather than clinically adjudicated diagnoses; therefore, the pattern is best understood as higher online engagement among women experiencing symptom/condition burden [26].

Economic gradients likely reflect differences in device access, data affordability, and digital skills, which are well-recognized determinants of general internet use among women in low- and middle-income settings. Population-level analyses in the Horn of Africa and beyond have linked higher household resources, education, and media exposure to greater connectivity and online health searches [33]. In Mogadishu, any such gradient is countered by rapid growth in connectivity. Industry compendia report that more than half of Somalia’s population was online at the start of 2025 and that social media identities number in the millions, creating the conditions for high uptake among urban ANC clients [7]. The heavy orientation toward social platforms in our sample has potential implications for maternal health programs and policymakers, though these must be interpreted strictly within the limits of this cross-sectional study, which did not measure digital literacy, misinformation exposure, privacy practices, source credibility, or any maternal care outcome. Social media communities can extend informational reach, provide normative guidance, and buffer isolation, with emerging trials and feasibility studies in sub-Saharan Africa showing that moderated WhatsApp groups and SMS-helpdesk models can feasibly support adherence to antenatal recommendations and prompt care-seeking for danger signs in pregnancy [16,34,35]. However, concerns regarding quality, safety, and equity are valid. App assessments and content audits repeatedly document wide variability in accuracy, sparse citation of sources, and inconsistent coverage of key topics, while many users report high confidence in what they read despite these issues. This underscores the classic “confidence–quality” gap and the need for health system engagement to curate trustworthy options [18,36,37]. The wider online environment shows similar variability: independent evaluations of topic-specific webpages relevant to pregnancy frequently find low credibility and readability challenges, which may disproportionately affect users with lower digital health literacy [38].

**Fig 4:**
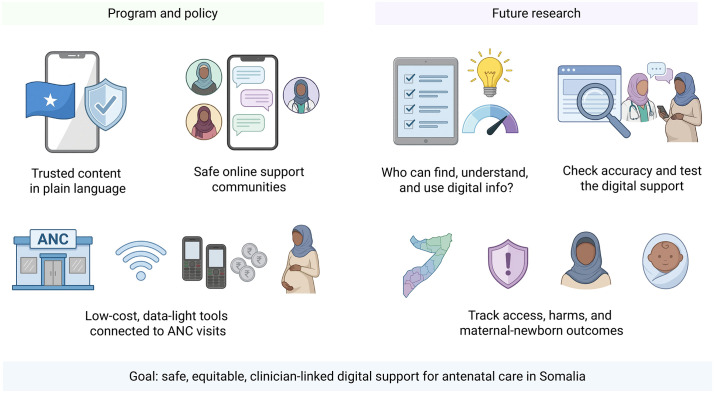
Program and research recommendations for safe digital pregnancy information in Mogadishu, Somalia. Created in BioRender. Ahmed, M. M. (2026) https://BioRender.com/urv1nna.

Therefore, our findings align with global guidance that positions digital tools as complements, not substitutes, for functioning maternal health systems. The WHO guidelines on digital health recommend targeted client communications, tele-advice, and digitized decision support where appropriate, but emphasize prerequisites such as content quality assurance, protection of privacy (Fig 4), and integration with services [39,40]. Evidence suggests that well-designed messaging and moderated social channels can improve information access and service uptake, particularly when linked to responsive clinical backstops [16,35]. For Mogadishu, such approaches are relevant, given the documented gaps across the continuum of care and the rapidly expanding digital footprint of women of reproductive age [7,41].

This study had several limitations. Because this was a cross-sectional study, it could not establish temporal order or causality; specifically, the study could not determine whether reported health problems prompted internet use or whether women who were already using online resources were more likely to recognize and report symptoms**.** Convenience sampling of ANC attendees in six clinics limits generalizability to pregnant women not in care, rural populations, and regions with weaker network coverage. In addition, because participation required literacy in Somali and completion of a self-administered questionnaire, women who could not read or write were excluded; this may have shifted the sample toward participants with relatively higher education and socioeconomic position and may have led to an overestimation of internet use compared with the broader pregnant population. All measures were self-reported, introducing risks of recall and social desirability bias and potential reporting uncertainty (including unknown/not disclosed responses). We did not independently verify the clinical conditions or the accuracy of the online sources used by the participants. Our study focused on frequency, channels, and topics rather than validated measures of eHealth or digital health literacy; prior studies suggest that literacy varies by socioeconomic status and may moderate the benefits and harms of online information use during pregnancy [42,43]. Although multivariable covariate selection used a liberal bivariable screening threshold (p < 0.25), residual confounding remains possible, and adjusted estimates should be interpreted cautiously**.** Because participants were recruited within six clinics, responses may be correlated within facilities and standard errors may therefore be underestimated. With only six clusters, cluster-robust standard errors, generalized estimating equations (GEE), and mixed-effects models are not reliable corrective options and were not applied; inference should therefore be interpreted cautiously. Given the common outcome prevalence, odds ratios may overstate effect magnitude relative to prevalence ratios; a modified Poisson sensitivity analysis was conducted to complement the primary AOR estimates. In addition, model-diagnostic measures were interpreted cautiously; with only six clinics, inferential precision and fit-related summaries may be less stable, so findings should be interpreted as association estimates rather than definitive predictive performance. The conceptual overlap between gravida and number of living children was addressed through sensitivity analyses with alternative model specifications, which confirmed stability of the primary findings. Finally, we did not evaluate privacy practices, data costs, or platform harm, all of which can shape women’s willingness and ability to engage online.

Programmatic implications should be interpreted in line with the study design and measurements. In this clinic-attending, literate ANC sample, we observed a high and frequent use of social media for pregnancy-related information. Because the data are cross-sectional and self-reported, we cannot infer the effects on ANC uptake, maternal-newborn outcomes, misinformation exposure, privacy harms, or causal direction for trimester and symptom/condition associations. Therefore, our recommendations are framed as hypotheses for future implementation research, not direct effect claims. Potential strategies to test include co-designed Somali-language moderated communities, verified micro-content on high-demand topics, referral pathways to clinician advice, adaptation of WHO digital intervention guidance to local ANC workflows, and equity-oriented access options (such as data-light content, zero-rated approved resources, and clinic-based connectivity), all evaluated with pragmatic designs in Mogadishu facilities. Future studies should include explicit privacy and governance testing for clinician-linked platforms, including privacy-by-design architectures (secure consent, role-based access control, end-to-end protection, auditable governance), with a potential evaluation of blockchain-supported personal data protection frameworks where contextually feasible [44]. To strengthen measurement, future Somali/regional studies should use validated digital health literacy tools (e.g., eHEALS, DHLI, and, where feasible, multidomain instruments such as eHLQ) [45–47], alongside resource-credibility audits and implementation trials that measure knowledge, care-seeking, service use, safety, and maternal-newborn outcomes.

## 5. Conclusion

Among pregnant women attending ANC in Mogadishu, internet use during pregnancy was common, frequent, and oriented toward social media and support-seeking. Patterns of engagement varied by socioeconomic and clinical context, with higher use among those in later gestation and those reporting current pregnancy health problems, with higher odds observed across income categories. These findings indicate that digital channels, particularly social platforms, are already part of women’s pregnancy information ecosystems. Health programs in Somalia may seek to build on this by aligning credible Somali-language content with antenatal counselling, establishing moderated peer communities linked to clinical advice, and setting standards for accuracy, privacy, and safety; These directions should be evaluated prospectively, since this study did not measure digital literacy, misinformation exposure, privacy practices, source credibility, or maternal care outcomes. Equity-focused measures, such as data-light resources, zero-rated access to approved content, and clinic-based connectivity options can help mitigate costs and access barriers. Future studies should also evaluate data protection and governance frameworks for maternal digital platforms (including clinician-linked and privacy-preserving models), alongside validated digital health literacy tools and resource quality audits, to determine whether integrated digital support improves knowledge, care-seeking, and maternal-newborn outcomes.

## Supporting information

S1 Table Comparison of adjusted odds ratios (AOR) from multivariable logistic regression and adjusted prevalence ratios (APR) from modified Poisson regression with robust standard errors for predictors of internet use for pregnancy-related information among pregnant women attending antenatal care in Mogadishu, Somalia (n = 422). (DOCX)

S2 Table Sensitivity analysis for primary predictors: adjusted odds ratios (AOR, 95% CI) for monthly income, gestational trimester, and health problem across the primary model and three alternative model specifications addressing collinearity between gravida and number of living children. (DOCX)
