# Supplementary material for: Internet use for pregnancy-related information and its correlates among women attending antenatal care in Mogadishu, Somalia
Source: PLOS Digit Health. 2026 Jul 30;5(7):e0001590. doi: 10.1371/journal.pdig.0001590 (PMC13423027; doi:10.1371/journal.pdig.0001590)
Supplement: S1 Table — (DOCX) [file pdig.0001590.s001.docx]

# S1 Table

Comparison of adjusted odds ratios (AOR) from multivariable logistic regression and adjusted prevalence ratios (APR) from modified Poisson regression with robust standard errors for predictors of internet use for pregnancy-related information among pregnant women attending antenatal care in Mogadishu, Somalia (n = 422).

| Variable | | Multivariable logistic regression | | | Modified Poisson regression (sensitivity) | |
| --- | --- | --- | --- | --- | --- | --- |
|  | **AOR (95% CI)** | | **p** | **APR (95% CI)** | | **p** |
| Monthly income (ref: <100 USD) |  | |  |  | |  |
| 101–300 USD | 2.16 | | 1.04–4.49 | 0.039 | | 1.25 |
| >300 USD | 3.99 | | 1.69–9.45 | 0.002 | | 1.39 |
| Gestational trimester (ref: First) |  | |  |  | |  |
| Second | 1.86 | | 0.96–3.61 | 0.067 | | 1.17 |
| Third | 2.55 | | 1.27–5.14 | 0.009 | | 1.25 |
| Health problem in pregnancy (ref: No) |  | |  |  | |  |
| Yes | 0.62 | | 0.32–1.22 | 0.168 | | 0.91 |
| Age group (ref: 18–25 years) |  | |  |  | |  |
| 26–35 years | 1.21 | | 0.67–2.22 | 0.528 | | 1.04 |
| ≥36 years | 1.49 | | 0.44–4.99 | 0.520 | | 1.08 |
| Education (ref: Primary) |  | |  |  | |  |
| No formal education | 0.86 | | 0.39–1.91 | 0.717 | | 0.96 |
| Secondary | 1.38 | | 0.76–2.51 | 0.289 | | 1.06 |
| University | 1.21 | | 0.56–2.61 | 0.627 | | 1.03 |
| Employment (ref: Housewife) |  | |  |  | |  |
| Employed | 1.02 | | 0.54–1.91 | 0.959 | | 1.01 |
| Student | 1.42 | | 0.72–2.79 | 0.314 | | 1.08 |
| Gravida (ref: Primigravida) |  | |  |  | |  |
| Multigravida | 1.82 | | 0.42–7.83 | 0.424 | | 1.15 |
| Number of living children (ref: No children) |  | |  |  | |  |
| 1–3 children | 0.61 | | 0.14–2.68 | 0.508 | | 0.89 |
| ≥4 children | 0.53 | | 0.11–2.61 | 0.438 | | 0.87 |
| Fetal sex (ref: Female) |  | |  |  | |  |
| Male | 0.54 | | 0.28–1.03 | 0.063 | | 0.89 |
| Prefer not to say/unknown | 0.58 | | 0.32–1.05 | 0.074 | | 0.90 |

AOR = adjusted odds ratio; APR = adjusted prevalence ratio; CI = confidence interval. Reference categories are shown in parentheses. Both models include the same covariates: monthly income, gestational trimester, health problem during pregnancy, age group, education level, employment status, gravida, number of living children, and fetal sex. The modified Poisson model uses robust (HC3) standard errors to account for the non-collapsibility of the Poisson variance in binary outcomes. The C-statistic for the primary logistic regression model was 0.689. The Hosmer–Lemeshow goodness-of-fit test for the primary model yielded χ² = 1.902, df = 8, p = 0.984, indicating adequate calibration.
