# Supplementary material for: Internet use for pregnancy-related information and its correlates among women attending antenatal care in Mogadishu, Somalia
Source: PLOS Digit Health. 2026 Jul 30;5(7):e0001590. doi: 10.1371/journal.pdig.0001590 (PMC13423027; doi:10.1371/journal.pdig.0001590)
Supplement: S2 Table — (DOCX) [file pdig.0001590.s002.docx]

**S2 Table**

Sensitivity analysis for primary predictors: adjusted odds ratios (AOR, 95% CI) for monthly income, gestational trimester, and health problem across the primary model and three alternative model specifications addressing collinearity between gravida and number of living children.

| Variable | Primary model  (all covariates) | Model 2a  (excludes gravida) | Model 2b  (excludes no. of children) | Model 2c  (excludes both) |
| --- | --- | --- | --- | --- |
| Monthly income: 101–300 USD | 2.16 (1.04–4.49)  p=0.039 | 2.13 (1.03–4.42)  p=0.042 | 2.11 (1.02–4.37)  p=0.044 | 2.12 (1.02–4.38)  p=0.044 |
| Monthly income: >300 USD | 3.99 (1.69–9.45)  p=0.002 | 3.97 (1.68–9.39)  p=0.002 | 3.92 (1.66–9.24)  p=0.002 | 3.93 (1.67–9.25)  p=0.002 |
| Trimester: Second | 1.86 (0.96–3.61)  p=0.067 | 1.83 (0.95–3.56)  p=0.073 | 1.84 (0.95–3.56)  p=0.071 | 1.84 (0.95–3.56)  p=0.071 |
| Trimester: Third | 2.55 (1.27–5.14)  p=0.009 | 2.57 (1.28–5.18)  p=0.008 | 2.59 (1.29–5.22)  p=0.008 | 2.58 (1.28–5.19)  p=0.008 |
| Health problem: Yes | 0.62 (0.32–1.22)  p=0.168 | 0.62 (0.32–1.22)  p=0.166 | 0.63 (0.32–1.22)  p=0.171 | 0.62 (0.32–1.21)  p=0.159 |

AOR = adjusted odds ratio; CI = confidence interval. All models include the same covariates as the primary model except where noted. Model 2a excludes gravida while retaining number of living children. Model 2b excludes number of living children while retaining gravida. Model 2c excludes both gravida and number of living children. Results are presented only for the three primary predictors of interest; full model results available on request.
